# The prevalence of potential alcohol–drug interactions in older adults

**DOI:** 10.3109/02813432.2013.788272

**Published:** 2013-06

**Authors:** Sirpa Immonen, Jaakko Valvanne, Kaisu H. Pitkälä

**Affiliations:** ^1^Espoo City Social and Health Services; Network of Academic Health Centers, University of Helsinki; Unit of General Practice, University Hospital of Helsinki, Helsinki, Finland; ^2^Tampere Medical School, University of Tampere, Tampere, Finland; ^3^Department of General Practice, University of Helsinki, and Unit of General Practice, Helsinki University Central Hospital, University of Helsinki, Helsinki, Finland

**Keywords:** Alcohol, alcohol–drug interaction, aged, drugs, Finland, general practice

## Abstract

**Objectives:**

The aim of this study was to assess the possibility of clinically significant drug–alcohol interactions among home-dwelling older adults aged ≥ 65 years.

**Design:**

This study was a cross-sectional assessment of a stratified random sample of 2100 elderly people (≥ 65 years) in Espoo, Finland. The response rate was 71.6% from the community-dwelling sample. The drugs were coded according to their Anatomical Therapeutic Chemical (ATC) classification index (ATC DDD 2012). Significant alcohol interactive (AI) drugs were examined according to the Swedish, Finnish, INteraction X-referencing (SFINX) interaction database, as well as concomitant use of central nervous system drugs, hypoglycaemics, and warfarin with alcohol. “At-risk alcohol users” were defined consuming > 7 drinks/week, or ≥ 5 drinks on a typical drinking day, or using ≥ 3 drinks several times/week, “moderate users” as consuming at least one drink/month, but less than 7 drinks/week, and “minimal/non-users” less than one drink/month.

**Results:**

Of the total sample (n = 1395), 1142 respondents responded as using at least one drug. Of the drug users, 715 (62.6%) persons used alcohol. The mean number of medications was 4.2 (SD 2.5) among “at-risk users”, 4.0 (SD 2.6) among “moderate users”, and 5.4 (SD 3.4) among “minimal/non-users” (p < 0.001). The concomitant use of AI drugs was widespread. Among the “at-risk users”, “moderate users”, and “minimal/nonusers” 42.2%, 34.9%, and 52.7%, respectively, were on AI drugs (p < 0.001). One in 10 of “at-risk users” used warfarin, hypnotics/sedatives, or metformin.

**Conclusions:**

Use of AI drugs is common among older adults, and this increases the potential risks related to the use of alcohol.

- The concomitant use of drugs with potential interactions with alcohol was common.
- Among the “at-risk users” and “moderate users”, 42.2% and 34.9% were on drugs potentially causing significant interactions with alcohol.
- It is important that clinicians discuss medication and alcohol consumption with their patients and warn patients who are prescribed alcohol interactive drugs about alcohol–drug interactions.

## Introduction

Over the past few decades, the use of drugs has increased among older adults [1,2]. Approximately nine in 10 of older adults take medications [1,3,4]. The number of drugs increases with advancing age [5]. Use of multiple medications increases the risk of adverse drug reactions (ADRs) and drug–drug interactions (DDIs) [6].

Although epidemiological studies have shown a decline in alcohol drinking along with age, the number of older adults drinking will increase when the age cohort born in the 1950s with their heavier drinking habits reach old age [7–9]. Use of alcohol has increased particularly among men and women aged between 65 and 74, and abstinence has decreased over the past decades [10]. According to our previous study [11], 8.2% of older adults drank in excess of the guidelines for persons ≥ 65 years [12].

Furthermore, many drugs interact adversely with alcohol [13–15]. Consistent with this, older adults identify interactions between alcohol and drugs as one reason to reduce their alcohol consumption [16]. However, many older adults concomitantly drink alcohol and take drugs. The ageing body is more susceptible to adverse drug and alcohol interactions; slower metabolic and clearance mechanisms delay their resolution [15]. Among a sample of adults 65–80 years of age, Onder et al. (2002) suggested that after adjusting for potential confounders, moderate alcohol consumption was associated with a 24% increase in the risk of an adverse drug reaction [17]. Some of these interactions are due to age-related changes in the absorption, distribution, and metabolism of alcohol and medications. Others are due to disulfiram-like reactions observed with some medications, exacerbation of therapeutic effects and adverse effect of medications when combined with alcohol, and interference of alcohol with effects of some medications. [15]. For example, warfarin has significant drug, food, and alcohol interactions and concomitant use of alcohol may increase or decrease warfarin metabolism [18]. It has high inter-individual and intra-individual variations in international normalized ratios (INR). Alcohol enhances the sedative effects of antidepressants, barbiturates, benzodiazepines, and opioids, creating the potential for adverse events such as falls [13,19], or cognitive decline or central nervous system depression [15]. Alcohol consumption by diabetic patients on hypoglycaemic agents can also increase the risk of hypoglycaemia [20]. Alcohol can potentiate the effects of metformin on lactate metabolism, which may rarely result in lactic acidosis, particularly in acute alcohol intoxication [21].

There are only a few epidemiological studies investigating the concomitant use of alcohol and drugs presenting a potential risk for older adults [4,17,19,22–24]. Of these studies, two examined emergency department or hospital admissions [17,19]. Thus, they are not representative of home-dwelling older populations. Two focused only on the concomitant use of alcohol and psychotropic drugs [23,24]. In a large population based US study, 77% of older people were exposed to potential alcohol–drug interactions [22]. In a previous study from Eastern Finland, the use of AI drugs among moderate drinkers was common [4]. The aim of this study was to assess the possibility of clinically significant drug–alcohol interactions among a random sample of home-dwelling older people aged 65 and older.

## Materials and methods

The data were gathered in May–September 2007. A postal questionnaire was sent to a stratified random sample of 2100 older individuals (≥ 65 years) from the Espoo Population Register. A stratified random sample of 350 was retrieved from each five-year age cohort (65–69 years, 70–74 years, 75–79 years, 80–84 years, 85–89 years, 90 years and over). The questionnaire was re-sent after three months to those who had not yet responded.

The questionnaire consisted of socio- demographic variables, health-related variables, and drinking patterns. Respondents were invited to list their medical diagnoses and medications prescribed by their doctors. The drugs were coded according to their Anatomical Therapeutic Chemical (ATC) classification index [25]. The Swedish, Finnish, INteraction X-referencing (SFINX) interaction database was used to assess the possibility of clinically significant drug–alcohol interactions (metronidazole J01XD01, tinidatzole J01XD02, disulfiram N07BB01, griseofulvin D01AA08, prazosin C02CA01, metformin A10BA02, tacrolimus D11AH01). In addition, this study investigated whether subjects had used central nervous system (CNS) drugs (antipsychotics N05A, antidepressants N06A, anxiolytics N05B, hypnotics N05C, antiepileptic drugs N03A, opioids N02A, hydroxyzine N05BB01), warfarin, or hypoglycaemics with alcohol.

Alcohol consumption was charted with several questions developed from the clinical guidelines for alcohol use in older adults [12] and the AUDIT [26]. Quantity and frequency were ascertained by asking: (i) “How often do you have a drink containing alcohol (a drink in Finland contains 12 g of alcohol), including any beer, cider, wine, or liquor; spirits?”, (ii) “On a typical day when you drink, how many drinks do you have? (1 drink = can or bottle (330 ml) of beer, 12 cl of wine, 4 cl of liquor; spirits (one shot glass), or 8 cl of sherry or madeira or aperitif)”, and (iii) “How often do you have three or more drinks on one occasion?”

We counted the amount of alcohol consumed by the respondent by taking into account the frequency of use and the portions consumed on one occasion. We defined “at-risk users” as those consuming > 7 drinks/week, or ≥ 5 drinks on a typical drinking day, or using ≥ 3 drinks several times/week to be in line with the guidelines for persons ≥ 65 years [12]. “Moderate users” were defined as consuming at least one drink/month, but less than 7 drinks/week, and “minimal/non-users” less than one drink/month.

Falls related to alcohol use were inquired about as follows: “Have you fallen or injured yourself when you have used alcohol (never/yes, but not during the last year/yes, during the last year)”.

We used the Statistical Package for the Social Sciences (SPSS) version 15.0 for Windows, and NCSS statistical analysis and graphics software to compute descriptive statistics. The “at-risk users”, “moderate users”, and “minimal or non-users” groups were compared with the chi-squared test in their categorical variables and the Kruskal–Wallis test was used for the non-normally distributed continuous variables.

## Results

### Description of respondents

Of the random sample, 31 had a native language other than Finnish or Swedish (inquiry form languages), 16 were deceased, 92 lived in permanent institutional care, and the postal address of 14 individuals had changed and was therefore unknown. Thus, the number of potential respondents was 1947, of whom 1395 returned the questionnaire. The response rate for the community-dwelling older persons was 71.6%.

Of the total sample (n = 1395), 1142 persons reported using at least one drug and had data available concerning their alcohol consumption. Their mean age was 78.7 years and 64.5% were females. By definition, 90 could be defined as “at-risk users”, 625 as “moderate users”, and 427 as “minimal/ non-users”. The “at-risk users” were more often males and married; they were younger and had higher education than the “non/minimal users”. The mean number of medications in these groups was 4.2 among “at-risk alcohol users”, 4.0 among “moderate users”, and 5.4 among “minimal/non-users” (p < 0.001) (Table I).

**Table I.** Characteristics of respondents with medication data (n = 1142) and their alcohol consumption.

Characteristic	At-risk users (n = 90)	Moderate users (n = 625)	Minimal or non-users (n = 427)	p-value^1^
Gender: male	76.7	39.5	20.8	< 0.001
Mean age	73.5	77.4	81.8	< 0.001
Marital status: Married or common-law marriage Widowed Single, unmarried, or divorced	74.0 14.4 11.5	58.7 29.0 12.2	38.0 47.9 14.1	< 0.001
Education: < 7 years 7–12 years > 12 years	14.4 43.3 42.2	26.1 52.6 21.3	39.0 46.0 15.0	< 0.001
Income: Good Moderate Poor	42.2 55.6 2.2	35.9 61.1 3.1	27.4 68.1 4.5	0.013
Self-reported health:				< 0.001
Healthy or quite healthy Unhealthy or very sick Charlson comorbidity index (SD)	80.0 20.0 1.0 (1.0)	81.9 18.1 0.8 (1.0)	62.0 38.0 1.2 (1.3)	< 0.001
Mean number of medications (SD)	4.2 (2.5)	4.0 (2.6)	5.4 (3.4)	< 0.001

Note: ^1^Differences between the groups in categorical variables were tested with a chi-squared test or Fisher's exact test and with non-normally distributed continuous variables with the Kruskall–Wallis test.

Participants had many diseases (Table II). The “minimal or non-users” usually had a higher number of diagnoses than the “at-risk” or “moderate” users. They also had poorer subjective health (see Table I).

**Table II.** Distribution of diagnoses among the groups using various amounts of alcohol (n = 1142).

	At-risk users (n = 90) %	Moderate users (n = 625) %	Minimal or non-users (n = 427) %	p-value^1^ Crude	Adjusted*
Prior myocardial infarction	5.6	5.4	12.1	0.005	0.010
Coronary heart disease	24.3	28.5	43.4	< 0.001	0.012
Hypertension	51.9	53.4	58.1	0.345	0.526
Prior stroke	1.5	2.3	5.8	0.034	0.0085
Asthma	16.9	15.2	21.2	0.127	0.314
Osteoarthritis	38.2	40.7	50.7	0.013	0.364
Diabetes Dementia	25.7 4.5	16.9 7.0	25.5 14.2	0.010 0.002	0.0087 0.262
Depression	10.8	13.5	23.3	0.001	0.089
Prior or current diagnosis of cancer	20.5	18.2	22.7	0.306	0.40

Notes: ^1^Differences between the groups were tested with a chi-squared test or Fisher's exact test. *Adjusted for gender and age.

### Prevalence of potential alcohol–drug interactions

Of the drug users, 62.2% (715) persons also used alcohol. The use of drugs with potential alcohol interactions among participants is presented in Table III. Among the “at-risk alcohol users” 42.2% were on AI drugs, whereas the respective figure among the “moderate users” was 34.9% and among the “non/minimal users” 52.7% (p < 0.001). The mean number of AI drugs in these groups was 0.67 (SD 0.98), 0.48 (SD 0.74), and 0.80 (SD 0.96), respectively. One in 10 of “at-risk users” and “moderate users” were on warfarin or hypnotics/sedatives (N05C). Of the groups, “at-risk users” used metformin more commonly than the other two groups (13.3% vs. 5.6% vs. 8.4%, p (adjusted) = 0.0089) (Table III).

**Table III.** Prevalence of use of some potential alcohol-interactive drugs among different alcohol consumption groups (n = 1142).

Medication	At-risk users (n = 90) %	Moderate users (n = 625) %	Minimal or non-users (n = 427) %	p-value^1^ Crude	Adjusted*
Metronidazole (J01XD01) Tinidatzole (J01XD02) Disulfiram (N07BB01) Griseofulvin (D01AA08) Prazosin (C02CA01) Tacrolimus (D11AH01) Antipsychotics (N05A) Antidepressants (N06A)	0 0 0 0 0 0 2.2 4.4	0 0 0 0 0.2 0 2.1 7.0	0.2 0 0 0 0.2 0 3.7 10.8	– – – – 0.88 – 0.252 0.038	– – – – – – 0.575 0.181
Anxiolytics (N05B)	6.7	3.7	7.5	0.022	0.053
Hypnotics/sedatives (N05C)	11.1	10.6	17.8	0.003	0.175
Antiepileptics (N03A)	5.6	1.8	3.3	0.062	0.091
Opioids (N02A) Hydroxyzine (N05BB01)	3.3 1.1	0.8 0.2	1.6 0.2	0.108 0.254	0.159 0.246
Warfarin (B01AA03)	11.1	9.3	15.7	0.007	0.024
Insulin (A10AB01)	1.1	2.6	3.5	0.392	0.144
Sulfonylureas (A10BB03)	6.7	3.8	6.8	0.084	0.040
Metformin (A10BA02)	13.3	5.6	8.4	0.015	0.0089

Notes: ^1^Differences between the groups were tested with a chi-squared test or Fisher's exact test. *Adjusted for gender and age.

Among the “at-risk users” on AI drugs, 13.8% reported they had fallen or injured themselves when using alcohol, whereas the respective figure among drug-users was 4.1% (p < 0.001).

## Discussion

### Summary

The concomitant use of drugs with potential interactions with alcohol was common. Of the drug users, 62.2% (715) persons also used alcohol. Among the “at-risk users”, “moderate users”, and “minimal/nonusers”, 42.2%, 34.9%, and 52.7% were on drugs potentially causing significant interactions with alcohol. Of the “at-risk users”, 11% were on warfarin, 11% on hypnotics/sedatives, and 13% on metformin, which is a serious concern for potential adverse events.

### Strengths and weaknesses

The strength of this study lies in the large and representative sample of older home-dwelling persons in bigger cities in Finland. The high response rate also supports its validity. However, relying on self-reporting measures imposes a limitation on the study since self-reported alcohol consumption is likely to be underestimated to some extent in drinking surveys [27–29]. The cross-sectional nature of this study limits our ability to fully explore the associations identified. Our data give a picture of the use of alcohol and potential AI drugs, but this does not allow us to evaluate whether alcohol and drugs are actually used concomitantly in everyday life and what kind of adverse events they expose these individuals to. The problems and adverse events related to alcohol– drug interactions depend on both quality and quantity of medication and quantity of alcohol used as well as the regularity and simultaneity of their consumption in the daily rhythm of life [13,14,17].

The “at-risk users” and “moderate users” were younger, healthier, and more often males and more educated than the “non-users”. The cross-sectional nature of our study does not allow us to evaluate whether older people decrease their consumption of alcohol when they get older, have more illnesses and receive more medications, or whether these findings reflect changing trends in lifestyle of older and younger cohorts. Some prior studies have suggested that the cohorts born later actually use more alcohol than the older cohorts [7–9].

### Potential adverse effects related to AIs

Falls and injuries were more common among “at- risk users” using AI drugs concomitantly than among others. Both alcohol and drugs with central nervous system effects can increase the risk of falls [30,31]. Alcohol and sedatives can reduce awareness and balance, which in turn can increase the risk of injurious falls [31]. Alcohol consumption and the use of psychotropic drugs have become more prevalent among older adults [3,23]. In our study, almost half of the users of antipsychotics, anxiolytics, hypnotics, and antidepressants drank alcohol. In a previous Finnish study, almost 40% of the users of antidepressants, benzodiazepines, sleeping pills, and opiates drank alcohol [4].

The metabolism of warfarin is influenced by alcohol. The use of warfarin may expose individuals to higher risk of serious bleeding [13,18], especially when falling. Alcohol consumption can result in dangerously high or insufficient warfarin activity depending on the patient's drinking habits [13]. Occasional consumption of low to moderate amounts of alcohol may not have an effect on warfarin anticoagulation whereas the effect of short-term consumption of large amounts of alcohol is unknown [32]. The effect of chronic use of large amounts of alcohol is less clear: it may prolong warfarin half- life without having an effect on an international normalized ration [32]. In our study of the “at-risk alcohol users”, 11% were on warfarin. Warfarin requires skilful dose management and patient communication to achieve the best outcomes. It is important that patients are aware of warfarin– alcohol interaction and their doctors of patients’ alcohol consumption.

Alcohol can potentiate the effects of metformin, which was the most commonly used oral hypoglycaemic in our study. Of the “at risk-users” 13.3% were on metformin, 6.7% used sulfonylureas, and 1.1% insulin. The use of metronidazole, tinidatzole, disulfiram, griseofulvin, prazosin, and tacrolimus was negligible.

### Clinical meaning of the study

The concomitant use of medications with potential interactions with alcohol was widespread. It is important that clinicians are screening the alcohol consumption (e.g. by using AUDIT) and discuss medication and alcohol consumption with their patients and warn patients who are prescribed alcohol interactive drugs about alcohol–drug interactions.

## References

[1] Haider SI, Johnell K, Thorslund M, Fastbom J (2007). Trend in polyfarmacy and potential drug–drug interactions across educational groups in elderly patients in Sweden for the period 1992–2002. Int J Clin Pharmacol Ther.

[2] Jyrkkä J, Enlund H, Korhonen MJ, Sulkava R, Hartikainen S (2009). Patterns of drug use and factors associated with polypharmacy and excessive polypharmacy in elderly persons: Results of the Kuopio 75 + Study: A cross-sectional analysis. Drugs Aging.

[3] Linjakumpu T, Hartikainen ST, Klaukka T, Koponen H, Kivela SL, Isoaho R (2002). Psychotropics among the home- dwelling elderly: Increasing trends. Int J Geriatr Psychiatry.

[4] Aira M, Hartikainen S, Sulkava R (2005). Community prevalence of alcohol use and concomitant use of medication: A source of possible risk in the elderly aged 75 and older?. Int J Geriatr Psychiatry.

[5] Jyrkkä J, Vartiainen L, Hartikainen S, Sulkava R, Enlund H (2006). Increasing use of medicines in elderly persons: A five-year follow-up of the Kuopio 75 + Study. Eur J Clin Pharmacol.

[6] Field TS, Gurwitz JH, Avorn J, McCormick D, Jain S, Eckler M (2001). Risk factors for adverse drug events among nursing home residents. Arch Intern Med.

[7] Sorocco KH, Ferrell SW (2006). Alcohol use among older adults. J Gen Psychol.

[8] Chan KK, Neighbors C, Gilson M, Larimer ME, Marlatt GA (2007). Epidemiological trends in drinking by age and gender: Providing normative feedback to adults. Addict Behav.

[9] Sulander T, Karisto A, Haarni I, Viljanen M (2009). Alcohol use and well-being: Preliminary research findings from baby boomers [Alkoholinkäytön ja hyvinvoinnin yhteyksiä. Alustavia tutkimustuloksia suurista ikäluokista] (in Finnish). Gerontologia.

[10] Laitalainen E, Helakorpi S, Uutela A (2008). Health behavior and health among Finnish elderly, spring 2007, with trends 1993–2007 (in Finnish).

[11] Immonen S, Valvanne J, Pitkälä KH (2011). Prevalence of at-risk drinking among older adults and associated sociodemographic and health-related factors. J Nutr Health Aging.

[12] National Institute on Alcohol Abuse and Alcoholism Helping patients who drink too much: A clinician's guide.

[13] Weathermon R, Crabb DW (1999). Alcohol and medication interactions. Alcohol Res Health.

[14] Adams WL, Gurnack AM, Atkinson R, Osgood NJ (2000). The effects of alcohol on medical illnesses and medication interactions. Treating alcohol and drug abuse in the elderly.

[15] Moore AA, Whiteman EJ, Ward KT (2007). Risks of combined alcohol/medication use in older adults. Am J Geriatr Pharmacother.

[16] Pringle KE, Heller DA, Ahern FM, Gold CH, Brown TV (2006). The role of medication use and health on the decision to quit drinking among older adults. J Aging Health.

[17] Onder G, Landi F, Vedova C, Atkinson H, Pedone C, Bernabei R (2002). Moderate alcohol consumption and adverse drug reactions among older adults. Pharmacoepidemiol Drug Saf.

[18] Camm AJ, Kirchof P, Lip G, Schotten U, Savelieva I, Ernst S (2010). Guidelines for the management of atrial fibrillation. Eur Heart J.

[19] Kurzthaller I, Wambacher M, Golser K, Sperner-Unterweger B, Haidekker A, Pavlic M (2005). Alcohol and benzodiazepines in falls: An epidemiological view. Drug Alcohol Depend.

[20] Krentz AJ, Ferner RE, Bailey CJ (1994). Comparative tolerability profiles of oral antidiabetic agents. Drug Safe.

[21] SFINX http://www.medbase.fi/sfinx/.

[22] Pringle KE, Ahern FM, Heller DA, Gold CH, Brown TV (2005). Potential for alcohol and prescription drug interactions in older people. J Am Geriatr Soc.

[23] Ilomäki J, Korhonen MJ, Enlund H, Hartzema AG, Kauhanen J (2008). Risk drinking behavior among psychotropic drug users in an aging Finnish population: The FinDrink study. Alcohol.

[24] Ilomäki J, Gnjidic D, Hilmer SN, Le Couteur DG, Naganathan V, Cumming RG, Waite LM, Seibel MJ, Blyth FM, Handelsman DJ, Bell JS (2012). Psychotropic drug use and alcohol drinking in community-dwelling older Australian men: The CHAMP study. Drug Alcohol Rev.

[25] ATC DDD (2012). WHO Collaborating Centre for Drugs Statistics Methodology. Anatomical Therapeutic Chemical (ATC) classification index.

[26] Babor TF, Higgins-Biddle JC, Saunders JB, Monteiro MG (2001). AUDIT. The alcohol use disorders identification test: Guidelines for use in primary care. WHO/MSD/MSB/016a.

[27] Hajat S, Haines A, Bulpitt C, Fletcher A (2004). Patterns and determinants of alcohol consumption in people aged 75 years and older: Results from the MRC trial of assessment and management of older people in the community. Age Ageing.

[28] Merrick EL, Horgan CM, Hodking D, Garnick DW, Houghton SF, Panas L (2008). Unhealthy drinking patterns in older adults: Prevalence and Aassociated characteristics. J Am Geriatr Soc.

[29] Sulander T (2005). Functional ability and health behaviors: Trends and associations among elderly people,1985–2003. Dissertation, Department of Epidemiology and Health Promotion, National Public Health Institute, Helsinki, Finland and Department of Social Policy University of Helsinki, Finland. Helsinki.

[30] Stenbacka M, Jansson B, Leifman AM, Romelsjö A (2002). Association between use of sedatives or hypnotics, alcohol consumption, or other risk factors and a single injurious fall or multiple injurious falls: A longitudinal general population study. Alcohol.

[31] Hartikainen S, Lönnroos E, Louhivuori K (2007). Medication as a risk factor for falls: Critical systematic review. J Gerontol A Biol Sci Med Sci.

[32] Cropp JS, Bussey HI (1997). A review of enzyme induction of warfarin metabolism with recommendations for patient management. Pharmacother.

